# Comparison of totally tubeless percutaneous nephrolithotomy and standard percutaneous nephrolithotomy for kidney stones: a randomized, clinical trial

**DOI:** 10.1590/1414-431X20154878

**Published:** 2016-03-18

**Authors:** N. Moosanejad, A. Firouzian, S.A. Hashemi, M. Bahari, M. Fazli

**Affiliations:** 1 Department of Urology, Mazandaran University of Medical Sciences, Sari, Iran; 2 Department of Anesthesia and Intensive Care, School of Medicine, Mazandaran University of Medical Sciences, Sari, Iran; 3 Faculty of Medicine, Immunogenetic Research Center, Mazandaran University of Medical Sciences, Sari, Iran; 4 Faculty of Medicine, Student Research Committee, Mazandaran University of Medical Sciences, Sari, Iran; 5 General Practitioner in Imam Khomeini Hospital of Esfarayen, Esfarayen Faculty of Medical Sciences, Esfarayen, Iran

**Keywords:** Totally tubeless percutaneous nephrolithotomy, Standard percutaneous nephrolithotomy, Complications

## Abstract

This study aimed to compare the totally tubeless percutaneous nephrolithotomy and standard percutaneous nephrolithotomy techniques regarding their rates of success and complications in patients with kidney stones. Patients were randomly assigned to two groups. Forty-four patients (24 men; mean age: 50.40±2.02 years) received totally tubeless percutaneous nephrolithotomy (PCNL; no nephrostomy catheter or ureteral catheter after PCNL) and 40 patients (18 men; mean age: 49.95±13.38 years) underwent standard PCNL (a nephrostomy catheter and ureteral catheter were used after PCNL). All surgeries were performed by one surgeon. Postoperative changes in hemoglobin, the blood transfusion rate, changes in creatinine levels, operation time, analgesic need, hospitalization time, and complication rate were compared between the groups. No significant differences were observed in age, gender, stone size, and surgery side between the groups (P<0.05). The operation time was significantly lower in the totally tubeless PCNL group than in the standard PCNL group (P=0.005). Pethidine requirements were significantly higher in the standard PCNL group than the totally tubeless PCNL group (P=0.007). Hospitalization time was significantly higher in the standard PCNL group than in the totally tubeless PCNL group (P<0.0001). The complication rate was 15% in the standard PCNL group and 9.1% in the totally tubeless PCNL group (P=0.73). The totally tubeless PCNL technique is safe and effective, even for patients with staghorn stones. This technique is associated with decreased pain, analgesic needs, and operative and hospitalization time. We believe that a normal peristaltic ureter is the best drainage tube.

## Introduction

Kidney stones are a common disease that affects at least 10% of people. A total of 70% of people who are affected by kidney stones experience recurring kidney stones (1). Various non-invasive, minimally invasive, and invasive methods have been reported as a treatment for kidney stones, including medicinal treatment, extracorporeal shock wave lithotripsy (ESWL), percutaneous nephrolithotomy (PCNL), and open renal surgery. In the past 30 years, PCNL as a minimally invasive method has been an effective treatment for large stones located in the kidney and upper ureter. PCNL is a more effective treatment for stones <2 cm compared with the ESWL method (1). PCNL is currently used for anomalies and in patients with ectopic pelvic kidneys, horseshoe kidneys, and malrotated kidneys, as well as in children and morbidly obese patients. PCNL is also used for calyceal diverticular calculi, upper calyceal calculi with infundibular stenosis, and a lower calyx >10 mm, which cannot be completely removed by ESWL (2 3 4 5). PCNL includes four steps: access to the kidney, dilatation of the tract (access site), nephroscopy and fragmentation of stones, and finally inserting a nephrostomy tube. Until 1997, the standard PCNL method used a nephrostomy catheter. After that, tubeless PCNL was introduced as a method to decrease complication rates; it is more practical and convenient, with a shorter hospitalization time. Furthermore, tubeless PCNL has decreased the amount of pain and the time needed to return to normal activities (6,7). In tubeless PCNL, the fourth step (i.e., inserting a nephrostomy tube) is not carried out (8). Recently, a more modern PCNL technique was introduced: totally tubeless PCNL. In this method, a nephrostomy catheter, a double J stent, or a ureteral catheter are not inserted after surgery (9 10 11 12). A ureteral stent can cause dysuria and pollakiuria. Removal of the stent at a later time results in increased complication rates (9). Therefore, this study aimed to investigate whether inserting a nephrostomy catheter and a ureteral catheter simultaneously results in a reduction of pain, hospitalization time, and postoperative complications.

## Patients and Methods

### Ethics

All of the patients gave written consent to participate in the study. This study was conducted in accordance with the Declaration of Helsinki and good clinical practice according to the International Conference on Harmonization guidelines. The ethics committee of Mazandaran University of Medical Sciences, Sari, Iran, approved this study. The study was designed as a randomized, clinical trial (IRCT: 201407256803N8).

### Inclusion and exclusion criteria

**Figure 1:**
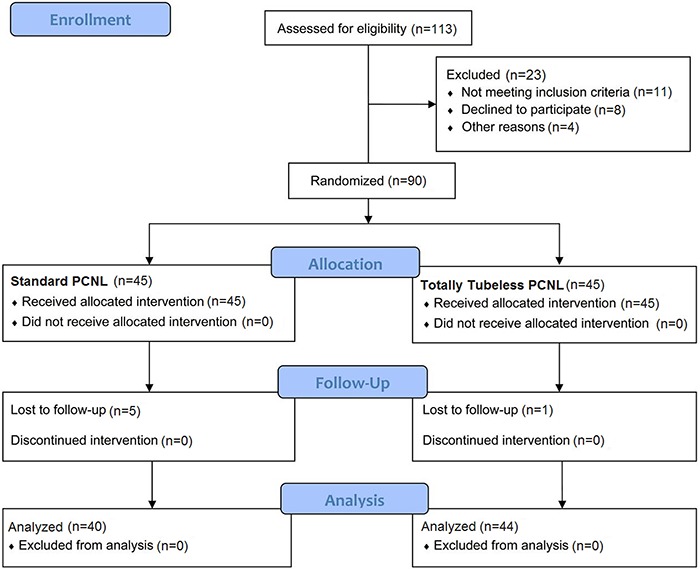
CONSORT flow diagram. PCNL: percutaneous nephrolithotomy.

The inclusion criterion was patients older than 20 years who were chosen for elective surgery of kidney stones via the PCNL technique. Exclusion criteria were patients with horseshoe kidneys, congenital renal anomalies, serious bleeding, perforation in the collecting system, and previously operated kidneys. Based on a study by Crook et al. (12) with a power of 80% and a significance level of 0.05, a sample size of 80 patients was required (n=80). Therefore, 113 patients were enrolled and divided into two groups (Figure 1).

### Methods of operations

After matching the patients in terms of age, gender, and underlying disease, they were randomized into two groups (Figure 1): 44 patients (24 men; mean age 50.40±2.02 years) were in the totally tubeless PCNL group and 40 patients (18 men; mean age 49.95±13.38 years) were in the standard PCNL group. Preoperative tests included measurement of hemoglobin levels, serum creatinine levels, and size and location of stones. Radiological evaluation was performed with ultrasonography, intravenous pyelography, and computed tomography scans. The patients received general anesthesia. The patients were placed in the lithotomy position to insert a ureteral catheter (4F-5F). They were then placed in the prone position. One access was performed in every patient. Access to the calyx was performed using a C-armed and 18-gauge needle. For entering the collecting system, a guide wire of 0.038 inches was inserted into the needle. With the aid of the guide wire, dilatation was performed with Amplatz dilators, and an Amplatz sheath (28F-30F) (Richard Wolf company, Germany) was placed (9,10). Stone fragmentation was carried out with a pneumatic lithotripter, and an X-ray was performed for residual stone fragments. At the end of the operation, if there was not any rupture of the renal pelvis, the Amplatz sheath was removed. After 4 or 5 h, the ureteral catheter and Foley catheter were removed in the totally tubeless PCNL group. Among this group, in any patients with residual stones, rupture, or major bleeding, a nephrostomy catheter was inserted and the patients were excluded from the study. In patients with upper calyceal stones, access to the upper calyx was performed through the middle calyx. Intraoperative extravasation was also performed in 6 patients in the totally tubeless PCNL group and in 4 patients in the standard PCNL group.

### Postoperative follow-up

On the first day following surgery, the patients were examined using plain abdominal X-rays and ultrasonography. Stone-free patients or patients with insignificant residual stones (<5 mm) were discharged from the hospital. Patients with stone fragments between 6 mm and 2 cm received a second ESWL procedure or PCNL. PCNL was performed in patients with stone fragments <2 cm. All preoperative tests, such as a complete blood count, urine culture, serum creatinine and urea levels, were repeated. Two months following surgery, plain abdominal X-ray and ultrasonography were performed. If there were no pathological findings, further examinations were not required. If extra calyceal anomalies, including hematoma or collection, were observed, a computed tomography scan was performed. However, if an anomaly was found inside the system (e.g., hydronephrosis), intravenous pyelography was carried out.

Age, gender, stone diameter, operation time (period of time between the beginning and end of the procedure), pre- and postoperative changes in hemoglobin, the complication rate, the blood transfusion rate, analgesic need, and hospitalization time were compared between the two groups (9).

### Data analysis

Data were analyzed by SPSS version 18.0 (SPSS, USA). To compare qualitative data, the chi-square test and Fisher's exact test were used as appropriate. The *t*-test was used to analyze quantitative data. A P value less than 0.05 was considered to be significant (9).

## Results

Eighty-four patients were enrolled in this study. Patients' preliminary and demographic data, including age, gender, surgery side, and stone size, are shown in Table 1. No significant differences were observed in age, gender, surgery side, stone size, and stone location between these two groups (Table 1, P>0.05). The mean operation time in the standard PCNL group (53.37±5.54 min) was significantly higher than in the totally tubeless PCNL group (50.32±3.83 min) (P=0.005; Table 2).

**Table:**
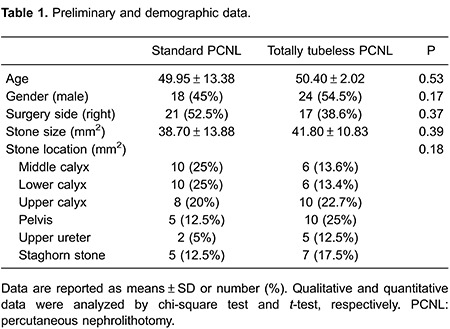

**Table:**
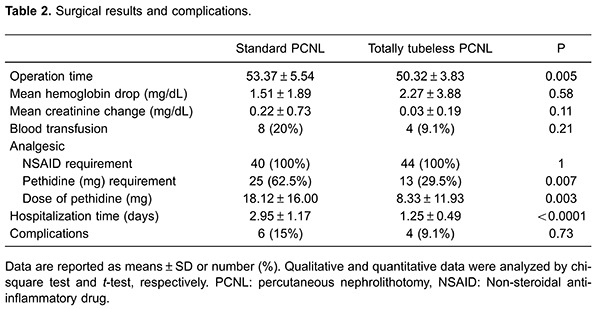

The mean preoperative hemoglobin level was significantly higher in the standard PCNL group (13.72±2.07 mg/dL) than in the totally tubeless PCNL group (12.88±1.45 mg/dL, P=0.038). The mean postoperative hemoglobin level was 11.81±2.25 mg/dL and 11.31±1.83 mg/dL in the standard PCNL and totally tubeless PCNL groups, respectively, with no significant difference between the groups (P=0.311). In general, the mean decline in hemoglobin level was 1.51±1.89 mg/dL and 2.27±3.88 mg/dL in the standard PCNL and totally tubeless PCNL groups, respectively, with no significant difference between the groups (P=0.58; Table 2).

The mean preoperative creatinine level was significantly higher (1.63±1.48 mg/dL) in the standard PCNL group than in the totally tubeless PCNL group (1.00±0.28 mg/dL, P=0.011). The mean postoperative creatinine level was also significantly higher in the standard PCNL group (1.28±0.54 mg/dL) than in the totally tubeless PCNL group (0.98±0.17 mg/dL, P=0.001). However, the mean decline in creatinine levels was not significantly different between the standard PCNL group and the totally tubeless PCNL group (0.22±0.73 mg/dL *vs* 0.03±0.19 mg/dL, P=0.11; Table 2).

Eight (20%) patients in the standard PCNL group and four (10%) patients in the totally tubeless PCNL received one blood transfusion during the procedure, with no difference between groups (P=0.21; Table 2).

When patients complained of pain, two types of analgesics were administered to the patients. First, a non-steroidal anti-inflammatory analgesic was provided. If this was not sufficient, a narcotic analgesic (pethidine) was administered. All of the patients were administered diclofenac. Twenty-five (62.5%) patients in the standard PCNL group and 13 (32.5%) in the totally tubeless PCNL group required pethidine. The mean dose of pethidine that was administered to the standard PCNL and totally tubeless PCNL groups was 18.12±16.00 and 8.33±11.93 mg, respectively. The need for pethidine (P=0.007) and the dose (P=0.003) administered to the totally tubeless PCNL group were significantly lower compared with those for the standard PCNL group (Table 2).

The mean hospitalization time was significantly lower in the totally tubeless PCNL group (1.25±0.49 days) than in the standard PCNL group (2.95±1.17 days, P<0.0001; Table 2).

Prolonged drainage, hematuria, and fever (body temperature >38.5°C) were observed in 6 (15%) patients in the standard PCNL group and in 4 patients in the totally tubeless PCNL group, with no significant difference between the groups (P=0.73). Prolonged renal colic and fever were observed in 2 patients in the totally tubeless PCNL group and in 2 patients in the standard PCNL group. Additionally, prolonged drainage, fever, and hematuria were observed in 4, 1, and 1 patient in the standard PCNL group, respectively. A patient with hematuria was diagnosed with pseudoaneurysm and administered tranexamic acid 250 mg at 6-h intervals.

## Discussion

In recent years, the PCNL technique has been improved several times to reduce pain, hospitalization time, and the rate of complications. Using small-caliber nephrostomy, an external ureteral stent, a double J stent, or avoiding nephrostomy drainage results in reduced postoperative pain and hospitalization time (13 14 15 16 17). This study aimed to compare totally tubeless PCNL and standard PCNL in patients with kidney stones.

In our study, there were no significant differences between the two techniques regarding the patients' age, gender, mean change in hemoglobin, mean change in creatinine levels, and blood transfusion. Similar to the findings in this study, Istanbulluoglu et al. (9) reported no significant differences in stone size, hemoglobin levels, and blood transfusion between totally tubeless PCNL and standard PCNL.

Previous studies have used various analgesics. Aghamir et al. (18), Shah et al. (19), and Sofer et al. (20) used morphine, diclofenac, and pethidine, respectively. In our study, diclofenac and pethidine were administered to patients, but the need for analgesics in the totally tubeless PCNL group was lower compared with that in the standard PCNL group, which is in accordance with previous studies (9,21,22). Shen et al. and Gonulalan et al. (23,24) reported that patients who had surgery with the standard PCNL technique experienced worse pain and required more postoperative narcotic analgesic than patients treated with tubeless techniques. In our study, avoiding the use of a nephrostomy catheter and double J stent appeared to lead to reduced pain and need for analgesics in the totally tubeless PCNL group.

Istanbulluogh et al. (9,22) reported that no significant differences were observed between standard PCNL and totally tubeless PCNL techniques. However, similar to a previous study (25), we showed that the operation time was significantly lower in the totally tubeless PCNL group compared with the standard PCNL group. The mean hospitalization time in the totally tubeless PCNL group was also significantly lower compared with the standard PCNL group. Similar previous studies reported that the mean hospitalization time was significantly lower in the totally tubeless PCNL group in comparison with standard and tubeless PCNL technique (9,18,21,22,25), which could be due to decreased pain, and avoiding insertion of a nephrostomy and ureteral catheter.

Previous studies have reported varying complication rates. Karami et al. (26) investigated 60 patients (n=60) who were equally divided into two groups. They reported that 2 (6.6%) patients in the totally tubeless PCNL group and 1 patient in the standard PCNL group were affected by urinary tract infections. Istanbulluoglu et al. (9) reported that complications were observed in 2 patients in the totally tubeless PCNL group (4.5%) (retroperitoneal hematoma, n=1; long-lasting renal colic, n=1) and in 6 patients in the standard PCNL group (13.3%) (prolonged urine drainage, n=5; long-lasting fever, n=1) (9). Another study by Istanbulluoglu et al. (22) showed complications in 2 patients in the standard PCNL group (4.6%) and in 7 patients in the totally tubeless PCNL group (7.6%). In the present study, complications were observed in 6 patients in the standard PCNL group (15%) (prolonged urine drainage, n=4; fever, n=1; pseudoaneurysm, n=1) and in 4 patients in the totally tubeless PCNL group (10%) (long-lasting renal colic, n=2; fever, n=2), with no significant difference between the groups. However, Gonulalan et al. (24) reported more frequent complications in the standard PCNL method in comparison with the tubeless technique.

In this study, 7 patients in the totally tubeless PCNL group and 5 patients in the standard PCNL group had staghorn stones. One patient in the standard PCNL group and one patient in the totally tubeless PCNL group experienced morbidity, but they did not require a secondary procedure, and there was no significant difference between the groups. These results are consistent with a study by Wang et al., (27), who reported that tubeless PCNL was a safe, efficacious, and cost-effective option in renal staghorn calculi. Additionally, tubeless PCNL was associated with low morbidity, short hospital stay, high stone-free rate, and early return to normal activity (27).

Our results showed that totally tubeless PCNL is a safe and effective technique and can be suggested for patients with staghorn stones. This technique is associated with decreased pain, analgesic need, operation time, and hospitalization time. We believe that a normal peristaltic ureter is the best drainage tube. However, a further study with a larger sample size is required to investigate the effectiveness of this technique in these patients.
